# Validation of Single-Camera MediaPipe BlazePose for Knee Joint Angle Measurement Across Multiple Functional Tasks: Concurrent Validity, Interrater Reliability, and Exploratory Test–Retest Reliability Study

**DOI:** 10.2196/102399

**Published:** 2026-11-03

**Authors:** Xiaotian Yue, YiXuan Gu, Bo Yu

**Affiliations:** 1 Department of Rehabilitation, School of International Medical Technology, Shanghai Sanda University, 2727 Jinhai Road, Pudong New District, Shanghai, 201209, China; 2 Orthopaedic Rehabilitation Unit, Department of Rehabilitation Medicine, Shanghai General Hospital, Shanghai Jiao Tong University School of Medicine, No. 100, Haining RoadShanghai, 200080, China, 86 18917946530

**Keywords:** markerless motion capture, pose estimation, MediaPipe BlazePose, knee kinematics, concurrent validity, interrater reliability, test–retest reliability, telerehabilitation, Kinovea, goniometry

## Abstract

**Background:**

Single-camera markerless pose estimation may support accessible knee movement assessment, but performance can depend on task, joint angle, and detection quality, and evidence across complementary measurement properties remains limited.

**Objective:**

This study aimed to evaluate agreement of MediaPipe BlazePose Heavy knee-flexion estimates with goniometer-set static target positions and with Kinovea 2D video annotation for dynamic tasks, to assess interrater agreement of the Kinovea annotations, and to explore repeated-session performance in a small retest subsample.

**Methods:**

Squat, sit-to-stand, and bilateral single-leg stance tasks were performed by 15 healthy adults during standardized lateral-view smartphone recording. Static knee flexion was assessed at 4 goniometer-set targets (30°, 60°, 90°, and 120°). Native hip-knee-ankle included angles were converted to clinical knee flexion (0° at full extension). Agreement was assessed using the intraclass correlation coefficient (ICC), specifically ICC_2,1_, signed bias and 95% limits of agreement, mean absolute error (MAE), and root mean square error (RMSE). Primary intervals used participant-cluster bootstrapping, and limits of agreement accounted for repeated measurements. Dynamic primary estimates were conditional on BlazePose target-frame confidence ≥0.3; low-confidence observations were also reported as detection failures and included in an all-output stress test. Four participants returned after 21 days to 29 days (median 25 d) for exploratory repeated-session analyses.

**Results:**

Dynamic measurement was available in 177 of 210 observations (84.3%); 33 of 210 observations (15.7%) failed the confidence criterion. Conditional task-specific ICCs were 0.773 (95% CI 0.564 to 0.875; n=71, 15 participants) for squat, 0.632 (95% CI 0.455 to 0.726; n=58, 12 participants) for sit-to-stand, and 0.136 (95% CI −0.078 to 0.482; n=48, 15 participants) for combined single-leg stance. Task-specific availability was present for 71 of 75 observations (95%), 58 of 75 observations (77%), and 48 of 60 observations (80%), respectively. Without the quality gate, squat ICC was 0.428, and RMSE was 25.43°. Static agreement with set targets resulted in an ICC of 0.874 (95% CI 0.770 to 0.953), with RMSE of 15.49° and angle-dependent signed error. Kinovea interrater ICC was 0.999 (95% CI 0.998 to 0.999) overall, which did not establish 3D accuracy. Repeated-session estimates with the 4 participants remained exploratory.

**Conclusions:**

With dynamic measurement available in 177 of 210 observations (84.3%), agreement among retained outputs depended on task, flexion angle, and detection quality. The findings support further feasibility and screening-level evaluation under controlled acquisition with per-recording quality checks; they do not support replacement of clinical goniometry, precise longitudinal decision-making, absolute 3D kinematic accuracy, or generalization to clinical populations or other smartphones.

## Introduction

### Background

Accurate measurement of knee joint kinematics is fundamental to the assessment and management of musculoskeletal conditions, including anterior cruciate ligament reconstruction recovery and knee osteoarthritis rehabilitation. Clinicians routinely monitor knee flexion and extension range of motion (ROM) to guide treatment progression, set discharge criteria, and evaluate surgical outcomes. In conventional clinical practice, the universal goniometer is used for static ROM measurement and offers acceptable intrarater reliability when used by trained clinicians [1]. However, the goniometer requires in-person contact, is limited to static or quasistatic postures, and cannot capture the dynamic joint behavior that characterizes functional tasks such as squatting, sit-to-stand transfers, and single-leg balance—tasks that more closely reflect the demands of daily living and return-to-activity benchmarks.

Laboratory-based, marker-based, motion-capture systems (eg, Vicon and Qualisys) are commonly used reference methods for 3D joint-kinematic analysis. However, they require dedicated space, specialized hardware, marker placement by trained personnel, and substantial postprocessing expertise [2,3]. These requirements limit their availability for routine or remote assessment, where neither an in-person goniometer nor laboratory motion capture is readily deployable.

The rapid development of markerless pose estimation technologies has created new possibilities for accessible, low-cost movement analysis. Deep learning–based frameworks can estimate joint positions from standard video without physical markers. Stenum et al [4] demonstrated the feasibility of automated gait analysis from single-camera 2D pose estimation. Among single-camera 2D approaches, Gupta et al [5] compared MediaPipe Pose with Kinovea 2D video annotation for knee flexion during gait, reporting a Pearson correlation of 0.941 and a mean absolute error (MAE) of 5.88°. Langley et al [6] compared both smartphone-based Deep Vision and Kinovea with 3D motion capture in patients with knee osteoarthritis. For Deep Vision, limits of agreement for peak flexion and ROM were within or equal to the study’s clinical thresholds. Koul et al [7] reported high intrarater reliability for knee flexion measurements using DETROM (intraclass correlation coefficient [ICC]=0.98) in healthy adults. Agreement with a universal goniometer was evaluated separately using Bland-Altman analysis. Akturk et al [8] applied MediaPipe to joint-angle and joint-moment estimation during sit-to-stand transfers, extending single-camera pose estimation to a functional task.

Multicamera and 3D markerless systems have also been evaluated during functional tasks. Lima et al [9] evaluated OpenCap, a multicamera system for 3D kinematic reconstruction, across functional tasks. For single-leg squatting, root mean square error (RMSE) ranged from 2.9° to 13.6° across joint angles. Peak-angle intersession ICCs across joints were 0.60 to 0.92 for single-leg squatting and 0.62 to 0.93 for the jump phase of a countermovement jump. Yoma et al [10] reported mean between-day root mean square differences <5° and mean ICCs of 0.77 to 0.83 across kinematic variables for Theia3D during single-leg squat and landing tasks, although hip flexion errors exceeded 5°. These findings provide context for single-camera studies, but differences in tasks, populations, and analysis methods limit direct performance comparisons.

Single-camera markerless measurements can be affected by self-occlusion and out-of-plane motion [3]. This study therefore focused on task-dependent agreement, the consistency of the 2D reference annotations, and repeated-session uncertainty. These measurement properties require evaluation before longitudinal or remote uses can be considered.

To our knowledge, few studies have evaluated an open-source single-camera pose-estimation system across multiple knee-related functional tasks in one cohort while examining complementary measurement properties, including agreement with a clinical instrument and a 2D video reference, interrater agreement of that 2D reference, and exploratory repeated-session performance. The selection and reporting of measurement properties were informed by COSMIN (Consensus-Based Standards for the Selection of Health Measurement Instruments) terminology [11].

### Objectives

The purpose of this study was to evaluate the MediaPipe BlazePose Heavy model for knee-flexion measurement in healthy young adults. We examined (1) interrater agreement of Kinovea 2D video annotation; (2) static agreement of BlazePose with goniometer-set target flexion across 4 angles; (3) dynamic agreement of BlazePose with Kinovea during squat, sit-to-stand, and bilateral single-leg stance; and (4) exploratory repeated-session agreement of BlazePose, Kinovea, and their between-method differences. We expected performance to vary by task, joint angle, and detection quality. This study did not evaluate absolute 3D kinematic accuracy, clinical populations, or cross-device equivalence.

## Methods

### Study Design

This cross-sectional validation study evaluated agreement of smartphone-based MediaPipe BlazePose knee-flexion estimates with goniometer-set targets for static positions and with Kinovea 2D video annotations by 2 trained raters for dynamic functional tasks. Secondary analyses assessed interrater agreement of the Kinovea annotations and exploratory repeated-session performance in a 4-participant subsample. Kinovea was treated as a 2D reference, not as an absolute biomechanical standard.

### Participants

We recruited 15 healthy adults (8 men, 7 women; mean age 20.8, SD 0.6, range 20‐22 years; mean height 171.1, SD 9.4 cm; mean weight 70.7, SD 15.7 kg; mean BMI 23.9, SD 3.4, range 18.8‐31.3 kg/m²) via convenience sampling from a university community. Inclusion criteria were age 18 years to 45 years, no current lower-extremity pain, and ability to perform a full-depth squat independently. Exclusion criteria included a history of lower-extremity surgery, current lower-extremity injury, or any musculoskeletal condition limiting knee range of motion. The study was approved by the Academic Ethics and Integrity Review Committee of Shanghai Sanda University (approval number 2026006). All participants provided informed consent prior to data collection. The sample size of 15 was determined by feasibility considerations (participant availability and resource constraints within a single-semester data collection window) rather than an a priori power calculation; this is acknowledged as a limitation, and the achieved precision of ICC estimates is reported via 95% CIs throughout.

A subset of 4 participants (S01–S04) returned for an exploratory second session 21 days to 29 days after their initial session (median 25 d). The second session used the same static and dynamic task protocol. The dataset comprised 15 initial-session datasets plus 4 repeated-session datasets, yielding 342 videos processed through the BlazePose pipeline (228 static and 114 dynamic). The primary cross-sectional static analysis used the 180 Session 1 trials from S01-S15; the 48 Session 2 static trials were reserved for the exploratory repeated-session analysis.

### Equipment and Setup

All videos were recorded with the same iPhone 17 Pro rear camera mounted on a tripod in a lateral view at knee height and 2.0 m from the participant. The device was selected because it was available and readily accessible to the research team; using one device throughout kept acquisition hardware constant. Recordings used 1920×1080 H.264 video. Participants S03-S15 and all repeated-session recordings were captured at 60 frames per second (approximately 23 Mbps), whereas S01-S02 were captured at 120 frames per second (approximately 43 Mbps) during the pilot phase. Codec profile, pixel format (yuv420p), and color space (BT.709) were verified with FFprobe. Floor markings standardized participant and camera position. The study did not compare smartphones, lenses, device-specific image processing, or frame rates.

We placed 3 small reflective markers on each participant’s test limb to support manual Kinovea annotation: the greater trochanter (hip), the lateral femoral epicondyle (knee), and the lateral malleolus (ankle). The BlazePose pipeline used MediaPipe’s native keypoint detection and did not rely on these external markers. An anterior superior iliac spine marker was placed during some pilot trials but was not used in the final analysis pipeline. Participants wore dark-colored tight-fitting trousers or compression pants and a short-cropped top and were barefoot. The background was a plain pale wall with even lighting and no harsh shadows.

### Data Collection Protocol

#### Static Validation

For static validation, participants were seated at 4 target knee-flexion angles (30°, 60°, 90°, and 120°). Target positions were set with a digital limb goniometer (GemRed model 82311‐00; transparent polycarbonate arms; manufacturer-stated resolution 0.1°, accuracy ±1°, and range 0°-180°). The fulcrum was placed at the lateral femoral epicondyle, the stationary arm was aligned with the greater trochanter, and the moving arm was aligned with the lateral malleolus. For each of the 3 trials at each target, the goniometer was reapplied, and the angle was reread. The limb was adjusted so that the displayed value, rounded to the nearest degree, equaled the target; floor tape served only as a positional guide. Thus, all recorded integer values equaled their targets after rounding, corresponding to displayed readings within approximately ±0.5° of the target at positioning, not exact independent measurements of the recorded posture. Positioning was performed by a physiotherapist (DPT) or a trained student assessor, and a research assistant (Rater B) verified the setup. This check was not an independent criterion measurement. The goniometer was removed before recording, and participants held the position for approximately 5 seconds. The angle was not remeasured after recording. There were 180 Session 1 trials (15 participants × 4 targets × 3 trials) and 48 additional trials from 4 retest participants. The comparison therefore assessed agreement with goniometer-set target positions, not simultaneous criterion validity.

#### Dynamic Validation

We selected 3 functional tasks to represent a range of clinically relevant knee movements and to align with prior markerless motion capture validation literature [4,12]: squat, sit-to-stand, and single-leg stance.

##### Squat

The participants performed 5 consecutive repetitions at a self-selected pace, with feet shoulder-width apart and arms extended forward for balance. This task produced the greatest clinical knee flexion and a clearly identifiable target frame.

##### Sit-to-Stand

The participants performed 5 consecutive sit-to-stand transitions from a standard-height chair (approximately 45-cm seat height) at a self-selected pace, with arms extended forward for balance.

##### Single-Leg Stance

The participants stood on 1 leg for 15 seconds per trial; 2 trials were performed per leg (left and right), yielding 4 single-leg stance trials per participant. The supporting (standing) leg was the annotated limb for both BlazePose and Kinovea measurements. Beginning with S03, participants were instructed to position the nonstance leg so that it did not occlude the supporting leg in the camera view.

##### Assessment

Each participant completed 6 dynamic videos: 1 squat video containing 5 repetitions, 1 sit-to-stand video containing 5 repetitions, and 4 single-leg stance videos. For squat and sit-to-stand, raters identified the frame of maximum clinical knee flexion, equivalent to the minimum native hip-knee-ankle included angle. For single-leg stance, raters selected a steady midstance frame and measured the supporting leg, which remained near full extension (approximately 0°-20° of clinical flexion). This produced 14 target-frame observations per participant (5 squat, 5 sit-to-stand, 2 left single-leg stance, and 2 right single-leg stance) and 210 observations across 15 participants. The right knee was the default measurement side for bilateral squat and sit-to-stand; the supporting limb was measured for each single-leg stance trial.

### Automated Pose Estimation: MediaPipe BlazePose

#### Overview

All videos were processed using MediaPipe BlazePose [13] via the PoseLandmarker Task API (mediapipe 0.10.33, Python 3.9.6) with the Heavy model (pose_landmarker_heavy.task). The model outputs 33 body landmarks per frame. Processing was configured in VIDEO running mode (frame-by-frame with temporal tracking) with a single-pose constraint (num_poses=1) and a default minimum pose-detection confidence of 0.5. All processing was performed on a local workstation (Apple M2 Max).

For each detected frame, the pipeline first computed the native 2D hip-knee-ankle included angle at the knee from pixel-coordinate vectors. Native-included angles approach 180° at full extension and decrease with flexion. For reporting and all analyses, this value was converted to clinical knee flexion as 180° − included angle, so that 0° represents full extension and larger values represent greater flexion. Right-knee landmarks 24, 26, and 28 and left-knee landmarks 23, 25, and 27 were used. Per-frame confidence was defined as the minimum visibility score of the hip, knee, and ankle landmarks. Unless otherwise specified, positive signed error denotes greater clinical flexion from BlazePose than from the comparator.

#### Detection Threshold Protocol Deviation

The default minimum pose detection confidence was 0.5. We reprocessed 5 static videos with insufficient detections at 0.3: S09_static_120deg_2, S10_static_120deg_2, S10_static_30deg_3, S04_retest_static_120deg_2, and S04_retest_static_120deg_3. This threshold reduction was a protocol deviation and was not applied to the remaining videos. The first 3 videos contributed to the Session 1 static analysis, and the latter 2 contributed to paired Session-2 static analyses.

The primary analyses retained the threshold-rescued videos. Sensitivity analyses repeated the Session 1 static analysis after excluding the 3 affected Session 1 videos and repeated the exploratory static repeated-session analysis after excluding the 2 affected pairs.

#### Automated Video-Minimum Extraction (Exploratory Use Only)

The pipeline also generated one automated video-minimum native-included angle per dynamic recording after excluding values below the implemented 10° anatomical-plausibility bound. This corresponds to the maximum reported clinical flexion after conversion. Raw-peak confidence was the minimum hip, knee, and ankle landmark visibility score at that automated video-minimum frame. These video-level automated minima were not used for the primary time-matched concurrent agreement analysis. They were used only in the exploratory n=24 all-output repeated-session stress test described in the following sections, without a raw-peak confidence gate.

#### Confidence-Based Frame Filtering

For static videos, frames with confidence ≥0.8 contributed to the filtered trial mean. For the primary dynamic BlazePose-versus-Kinovea comparison, the BlazePose clinical flexion value at the Kinovea-selected target time was classified as a detection failure when target frame confidence was <0.3. This criterion yielded 33 failures among 210 available observations; the conditional primary agreement analysis used the remaining 177. An all-output sensitivity stress test retained all 210 numeric outputs to characterize performance in the absence of this quality gate; it was not treated as a replacement for the conditional analysis. The target frame cutoff was established during analysis, not prespecified before data analysis. The April 16 interim analysis of S01-S06 included all 84 observations; the April 27 full-sample script introduced the 0.3 cutoff as an operational flag for low-confidence pose estimation failure. No calibration nor documented rationale for the numerical cutoff was available. It was distinct from the 0.3 pose detection parameter used to reprocess 5 static videos. A stricter target frame cutoff of ≥0.5 was also examined as a sensitivity analysis.

### Kinovea 2D Video Reference

Two trained raters independently annotated task-specific target frame knee angles using Kinovea 2025.2. Each rater selected maximum clinical knee flexion for squat and sit-to-stand and a steady midstance frame for single-leg stance then digitized the greater trochanter, lateral femoral epicondyle, and lateral malleolus using the reflective markers as guides. The native 2D-included angle was converted to clinical knee flexion as 180° − included angle. Kinovea served as a 2D video-analysis reference; it was not assumed to provide absolute 3D joint kinematics.

Raters were blinded to each other’s annotations and to BlazePose output. Their mean clinical flexion angle was used as the Kinovea 2D reference for dynamic agreement, and their mean target frame time stamp was used for BlazePose time matching. High interrater agreement reduces uncertainty from rater inconsistency but does not remove systematic 2D reference error.

### BlazePose–Kinovea Time Matching

For the primary dynamic comparison, BlazePose was evaluated at the Kinovea-selected target time rather than at BlazePose’s automated video minimum. For each of the 210 observations, the mean of the 2 rater time stamps was calculated, and the closest time stamp in the BlazePose per-frame output was selected. The native-included angle at that frame was converted to clinical flexion before analysis. This separates target-time selection by the 2D reference from BlazePose angle estimation at that time.

### Descriptive Data Quality Grading

Each of the 342 videos received a descriptive A-D quality assurance grade based on the proportion of frames with confidence ≥0.8: Grade A (≥80%), Grade B (50%‐79%), Grade C (20%‐49%), or Grade D (<20%). These trial-level labels did not determine inclusion, weighting, or statistical inference and are not equivalent to target frame detection success. For a post hoc descriptive acquisition sequence summary, participants were grouped as S01-S06, S07-S10, and S11-S15; these groups were used only to describe target frame confidence and failure rates, without inferential testing. Detailed grade and detection quality distributions are reported in Tables S1-S5 in Multimedia Appendix 1 .

### Statistical Analysis

The original analyses used Python 3.9.6, pingouin for ICCs, and scipy.stats. The revision-stage participant cluster analyses used Python 3.12.14, NumPy 2.3.5, pandas 2.2.3, and SciPy 1.16.2. ICC_2,1_ was recomputed from the same ANOVA formula and verified against the frozen results. Figures were generated using matplotlib.

#### Concurrent Validity

Agreement was summarized using the 2-way random effects, single-measure, absolute agreement ICC, ICC_2,1_. Signed bias and limits of agreement (LoA) used the Bland-Altman framework [14]; MAE and RMSE quantified error magnitude. Static bias was BlazePose minus goniometer target clinical flexion, dynamic bias was BlazePose minus Kinovea clinical flexion, and interrater bias was Rater A minus Rater B. Pearson *r* described association not absolute agreement. ICC interpretation followed Koo and Li [15].

Primary cross-sectional inference retained the observation-level ICC and error estimands. We calculated percentile 95% CIs by resampling eligible participants with replacement, retaining all paired observations from each sampled participant (10,000 resamples; master seed 20260923; stable analysis-specific seeds). No trials were sampled independently. Dynamic agreement remained conditional on target frame confidence ≥0.3: Squat and combined single-leg stance each had 15 contributing participants, whereas sit-to-stand had 12. Participants with no retained observations remained in availability denominators. Repeated-measures LoA were calculated as bias ±1.96 times the square root of the sum of within- and between-participant variance components, allowing unequal repeats [16]. Negative estimated between-participant variance components were set to zero. Bootstrap CIs were also computed separately for bias and each LoA endpoint (Tables S16-S19 in Multimedia Appendix 1). Task-specific dynamic and angle-specific static results guided interpretation; pooled results are descriptive mixtures. Participant-mean analyses are supplementary and estimate agreement after averaging, not single-observation agreement. Participant-by-task or participant-by-angle cells from the same individual were not treated as independent for primary inference. Four-participant repeated-session results retained unadjusted descriptive intervals and were not used for primary inference.

For static analysis, the filtered-mean native BlazePose angle was converted to clinical flexion and compared with the goniometer target. Angle-specific results were reported to characterize error across 30°, 60°, 90°, and 120° of target flexion. For dynamic analysis, time-matched BlazePose clinical flexion was compared with the mean Kinovea clinical flexion value. Squat, sit-to-stand, and combined single-leg stance estimates were the main task-level results; side-specific single-leg stance and pooled cross-task estimates were secondary descriptive results.

#### Interrater Reliability

Interrater agreement of the Kinovea 2D annotations was assessed across 210 paired clinical flexion measurements using ICC_2,1_, bias and 95% LoA, MAE, RMSE, and Pearson *r*. Estimates were reported overall and by task. The a priori descriptive benchmark for acceptable interrater ICC was 0.90. Between-task significance tests were not performed because trial-level implementations would not account for repeated observations within participants.

#### Exploratory Repeated-Session Analyses

Four participants (S01-S04) completed a second session after observed intervals of 21 days to 29 days (median 25 d). All repeated-session analyses were exploratory and addressed distinct measurement questions rather than constituting a single reliability claim.

First, BlazePose repeated-session agreement was evaluated for 48 paired static trial means and, as an all-output stress test, for 24 paired automated video-minimum dynamic values (4 participants × 6 videos). The dynamic estimand used 1 automated video minimum per recording rather than a Kinovea-selected time-matched frame and applied no raw-peak confidence gate; 10 of the 24 pairs had at least one raw-peak confidence value <0.3.

Second, repeated-session agreement of the Kinovea 2D reference was explored using 56 paired task/trial clinical flexion observations (4 participants × 14 observations).

Third, conditional BlazePose-versus-Kinovea agreement was described for Session 2. The same target-frame confidence criterion used in Session 1 retained 35 of 56 available observations. This 4-participant analysis was not treated as a replication study.

Fourth, the between-method clinical flexion difference was calculated in each session for 34 observations valid at both sessions. The ICC of these paired differences was used as an exploratory indication of whether the observed between-method offset might be consistent across sessions. Given the 4-participant sample and repeated observations, the estimate was not interpreted as proof of either stability or instability.

Repeated-session analyses used ICC_2,1_, signed bias and 95% LoA, MAE, and RMSE. The analysis unit for these descriptive calculations was the repeated task/trial observation nested within 4 participants; the F-distribution ICC intervals did not adjust for participant-level clustering. Estimates were therefore presented with their denominators and uncertainty and were not used as headline clinical evidence.

#### Interpretation Thresholds

ICC values were described using the categories from Koo and Li [15]: <0.50, poor; 0.50‐0.75, moderate; 0.75‐0.90, good; and >0.90, excellent. Following Langley et al [6], ±5° for signed mean difference and ±10° for LoA were used only as descriptive contextual anchors for those same metrics, not as universal clinical acceptability thresholds. MAE and RMSE were reported as error-magnitude measures without assigning them a universal cutoff. Statistical association and agreement were interpreted separately, and no multiplicity-adjusted confirmatory inference was claimed for exploratory strata.

### Ethics Approval

This study was approved by the Academic Ethics and Integrity Review Committee of Shanghai Sanda University (approval number 2026006, April 14, 2026). The study was conducted in accordance with the Declaration of Helsinki. All participants provided informed consent prior to enrollment.

## Results

### Participants

The cross-sectional analysis included 15 healthy adults (8 men, 7 women; mean age 20.8, SD 0.6, range 20‐22 years; mean height 171.1, SD 9.4 cm; mean weight 70.7, SD 15.7 kg; mean BMI 23.9, SD 3.4, range 18.8‐31.3 kg/m²). All 15 participants completed the full data collection protocol without reported adverse events. Four participants (S01-S04; 2 men, 2 women) returned for the exploratory repeated-session assessment after 21 days to 29 days (median 25 d).

For the primary cross-sectional analyses, 180 static trials (15 participants × 12 conditions) and 210 dynamic Kinovea-annotated target-frame observations were available.

Figure 1 summarizes the task-specific interrater agreement, angle-specific static signed error, and task-specific conditional dynamic agreement with detection failure rates. Detailed results are presented in Tables 1–5 and Multimedia Appendix 1.

**Figure 1.**
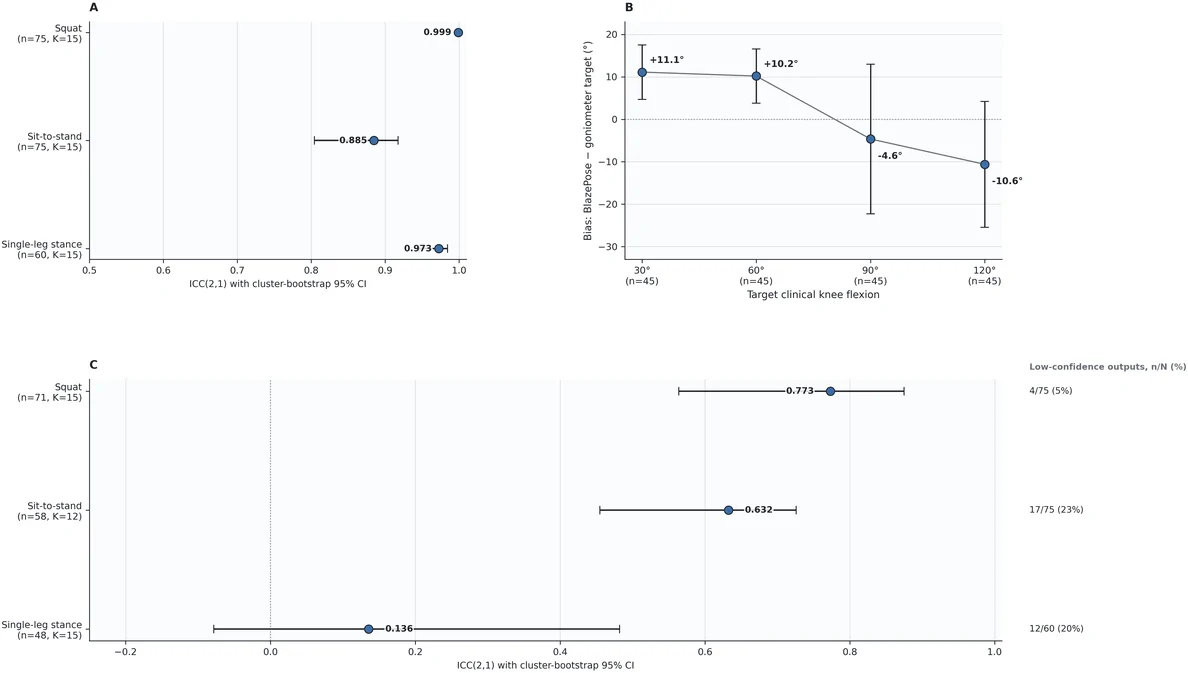
Task- and angle-specific measurement results: (A) task-level interrater reliability between Kinovea Raters A and B, with points showing the intraclass correlation coefficient (ICC)_2,1_, horizontal bars showing participant-cluster bootstrap 95% CI, K indicating the number of eligible participants, and n indicating the number of observations; (B) static BlazePose bias relative to goniometer target flexion, with points showing mean bias and error bars showing ±1 SD of paired differences not CIs and positive values indicating greater flexion estimated using BlazePose; (C) task-level dynamic agreement with the Kinovea 2D reference, conditional on target-frame BlazePose confidence ≥0.3, with the adjacent column reporting low-confidence outputs using all attempts as denominators. Single-leg stance combines left and right sides. All angles are clinical knee flexion, with 0° at full extension.

**Table 1.** Analysis domains, available and analyzed observations, and independent participants.

Analysis^a^	Comparison/estimand	Available observations, n	Analyzed observations, n	Independent participants, n	Role
Static agreement	BlazePose versus goniometer target	180	180	15	Main cross-sectional
Dynamic conditional agreement^b^	BlazePose versus Kinovea 2D reference; target-frame confidence ≥0.3	210	177	15 overall; 12‐15 by task^c^	Main cross-sectional; task-specific results lead
Interrater agreement	Kinovea Rater A versus Rater B	210	210	15	Main cross-sectional
BlazePose static repeated-session agreement	Session 1 versus Session 2	48	48	4	Exploratory
BlazePose dynamic video-minimum stress test^d^	Session 1 versus Session 2; automated video minimum, no confidence gate	24	24	4	Exploratory stress test
Kinovea repeated-session agreement	Session 1 versus Session 2	56	56	4	Exploratory
Session 2 conditional agreement^e^	BlazePose versus Kinovea 2D reference	56	35	4	Exploratory
Between-method difference across sessions	Session 1 versus Session 2 between-method differences	56	34 valid in both sessions	4	Exploratory

a Confidence interval and repeated measures LoA methods for cross-sectional analyses are described in the Statistical Analysis section and Tables S16-S19 in Multimedia Appendix 1.

b Excluded 33 of the 210 target-frame observations with BlazePose confidence <0.3 and reported them separately as detection failures.

c Primary dynamic cluster counts are 15 for squat, 12 for sit-to-stand, and 15 for combined single-leg stance.

d All-output automated video-minimum stress test; 10 of 24 pairs had at least one raw-peak confidence value <0.3.

e Retained 35 of the 56 observations.

**Table 2.** Interrater agreement of Kinovea 2D clinical knee-flexion annotations (n=210), with all angles representing clinical knee flexion (0° at full extension).

Task	Observations, n	Bias^a^	95% LoA^b^^,^^c^	MAE^d^	RMSE^e^	Pearson *r*^f^	ICC^g^^,^^h^_2,1_ (95% CI^i^)
Overall^j^	210	+1.15°	−3.51° to +5.81°	1.56°	2.62°	0.999	0.999 (0.998 to 0.999)
Squat	75	+0.09°	−1.25° to +1.43°	0.39°	0.68°	0.999	0.999 (0.997 to 0.999)
Sit-to-stand	75	+2.65°	−3.78° to +9.08°	3.13°	4.16°	0.936	0.885 (0.804 to 0.918)
Single-leg stance	60	+0.58°	−1.88° to +3.05°	1.05°	1.37°	0.977	0.973 (0.946 to 0.984)

a Bias: Kinovea Rater A clinical flexion−Rater B clinical flexion.

b LoA: limits of agreement.

c LoA use repeated-measures variance components; endpoint CIs are in Table S17 in Multimedia Appendix 1.

d MAE: mean absolute error.

e RMSE: root mean square error.

f Describes the association, not absolute agreement.

g ICC: intraclass correlation coefficient.

h 2-way random-effects, single-measure, absolute-agreement ICC.

i ICC CIs use participant-cluster bootstrapping (15 participants per row).

j Secondary descriptive cross-task summary.

**Table 3.** Static agreement of BlazePose clinical knee flexion with goniometer target flexion (n=180; S01-S15).

Target flexion	Observations, n	Bias^a^	95% LoA^b^^,^^c^^,^^d^	MAE^e^	RMSE^f^	SD(diff)^g^	SEM^h^^,^^i^^,^^g^
Overall^j^^,^^k^	180	+1.51°^l^	−28.85° to +31.87°	10.01°	15.49°	15.46°	10.93°
30°	45	+11.11°	−1.73° to +23.94°	11.15°	12.79°	6.41°	4.53°
60°	45	+10.18°	−2.66° to +23.03°	10.82°	11.99°	6.40°	4.53°
90°	45	−4.64°	−39.36° to +30.07°	7.45°	18.02°	17.60°	12.45°
120°	45	−10.60°	−39.71° to +18.50°	10.60°	18.10°	14.84°	10.49°

a Bias: BlazePose clinical flexion−goniometer target flexion.

b LoA: limits of agreement.

c LoA endpoint CIs are in Table S17 in Multimedia Appendix 1.

d LoA use repeated-measures variance components (15 participants per row).

e MAE: mean absolute error.

f RMSE: root mean square error.

g SD(diff) and SEM remain descriptive trial-level summaries.

h SEM: standard error of measurement.

i SEM =SD(diff)/√2.

j Overall ICC_2,1_=0.874 (95% CI 0.770 to 0.953).

k The ICC CI uses participant-cluster bootstrapping.

l Overall pooled bias is descriptive because angle-specific errors reverse direction.

**Table 4.** Conditional agreement of BlazePose with the Kinovea 2D clinical flexion reference at target frames with BlazePose confidence ≥0.3, with all values reflecting clinical knee flexion.

Task	Retained observations, n	Available observations, n	Failures from the available observations, n (%)	Bias^a^ (95% LoA^b^^,^^c^)^d^	MAE^e^	RMSE^f^	Pearson *r*^g^	ICC^h^_2,1_ (95% CI^i^)
Squat (n=15 participants)	71	75	4 (5)	+6.17° (−6.45° to +18.78°)	7.10°	8.78°	0.885	0.773 (0.564 to 0.875)
Sit-to-stand (n=12 participants)	58	75	17 (23)	+6.43° (−1.82° to +14.67°)	6.68°	7.59°	0.896	0.632 (0.455 to 0.726)
Single-leg stance, both legs combined (n=15 participants)	48	60	12 (20)	+2.01° (−23.42° to +27.44°)	7.51°	12.88°	0.173	0.136 (−0.078 to 0.482)
Overall, secondary descriptive (n=15 participants)	177	210	33 (15.7)	+5.12°^j^ (−11.30° to +21.55°)	7.07°	9.73°	0.987	0.982 (0.967 to 0.992)

a Bias: BlazePose−Kinovea 2D reference; positive values indicate greater flexion from BlazePose.

b LoA: limits of agreement.

c LoA use repeated-measures variance components.

d Bias and LoA endpoint CIs are in Tables S16-S19 in Multimedia Appendix 1.

e MAE: mean absolute error.

f RMSE: root mean square error.

g Describes an association, not absolute agreement.

h ICC: intraclass correlation coefficient.

i ICC CIs use participant-cluster bootstrapping.

j The overall pooled ICC is range-sensitive and is not a headline result.

**Table 5.** Exploratory repeated-session analyses with 4 participants (S01-S04; observed interval 21‐29 d, median 25 d); pooled estimates may be influenced by between-task or between-angle range and are not headline clinical evidence.

Exploratory analysis	Observations, n	Bias definition	Bias (95% LoA^a^)	MAE^b^	RMSE^c^	ICC^d^_2,1_ (95% CI)^e^
BlazePose static, Session 1 versus Session 2	48	Session 2−Session 1	−7.30° (−42.65° to +28.04°)	10.32°	19.28°	0.782^f^ (0.617 to 0.877)
BlazePose dynamic automated video-minimum stress test^g^	24	Session 2−Session 1	+18.92° (−82.04° to +119.89°)	34.71°	53.86°	0.522 (0.174 to 0.757)
Kinovea 2D reference, Session 1 versus Session 2	56	Session 2−Session 1	+1.19° (−12.05° to +14.42°)	5.63°	6.80°	0.991 (0.985 to 0.995)
BlazePose versus Kinovea, Session 2 conditional	35	BlazePose−Kinovea	+8.10° (−14.69° to +30.89°)	8.95°	14.03°	0.969 (0.889 to 0.988)
Between-method difference, Session 1 versus Session 2	34	Session 2 difference−Session 1 difference	+2.45° (−14.01° to +18.91°)	5.19°	8.63°	0.094 (−0.225 to 0.405)

a LoA: limits of agreement.

b MAE: mean absolute error.

c RMSE: root mean square error.

d ICC: intraclass correlation coefficient.

e Do not adjust for participant-level clustering.

f Excluding the 2 retest threshold-rescued static pairs changed the static repeated-session ICC from 0.782 to 0.766.

g Uses 1 automated video minimum per recording, applies no raw-peak confidence gate, and includes 10 of 24 pairs with at least one raw-peak confidence value <0.3.

### Interrater Agreement of Kinovea 2D Annotations

Overall interrater agreement for the Kinovea 2D clinical flexion annotations was high (ICC_2,1_=0.999, cluster-bootstrap 95% CI 0.998 to 0.999; MAE=1.56°; n=210 observations from 15 participants; Table 2; Figure 2C). This indicates consistent annotation between these 2 raters but does not establish absolute accuracy against 3D kinematics.

**Figure 2.**
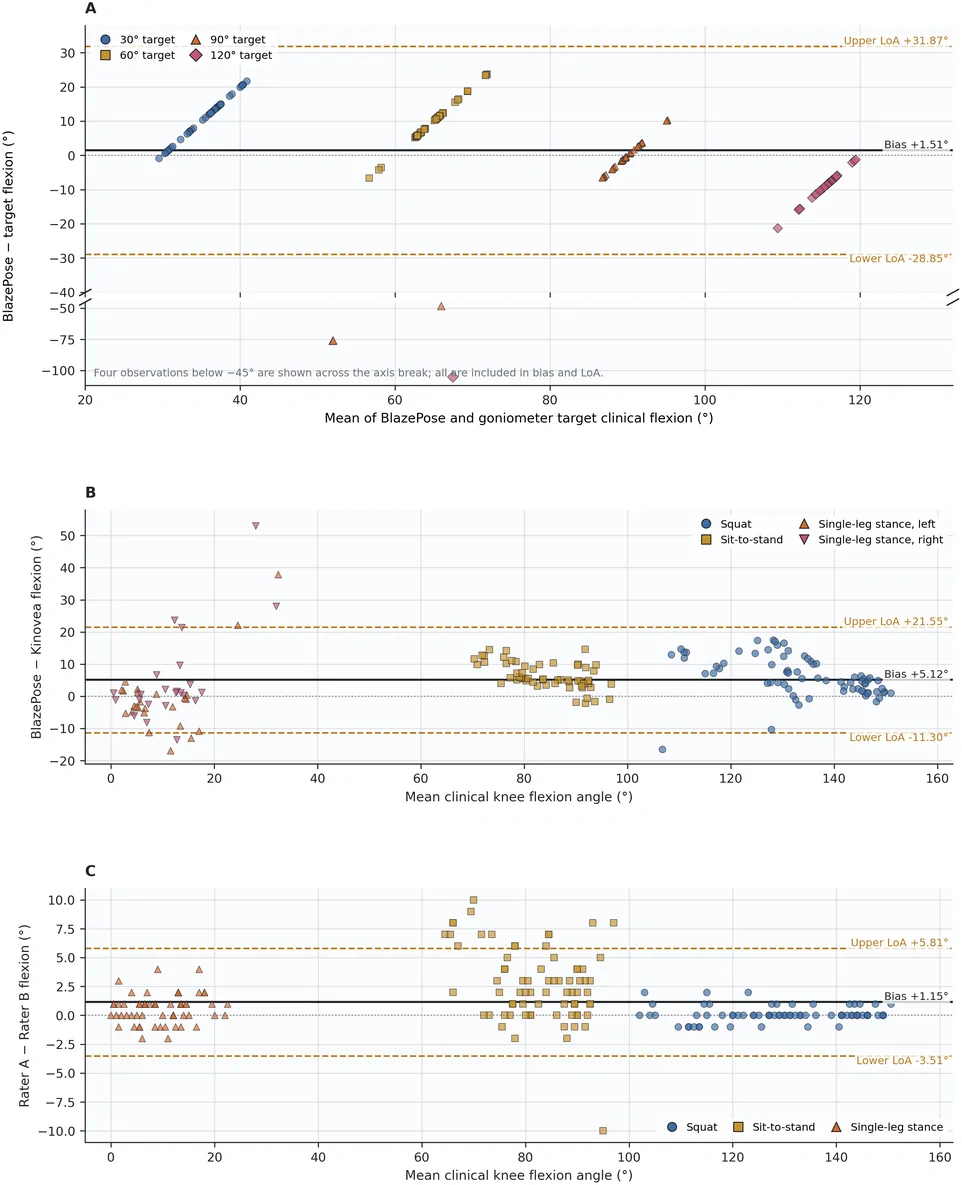
Bland-Altman plots using clinical knee flexion angles and showing the mean of paired angles on the x axis and the first-named measurement minus the second on the y axis, with positive differences indicating greater flexion from the first measurement: (A) BlazePose versus goniometer-set targets (n=180, 15 participants; bias +1.51°; 95% limits of agreement [LoA] –28.85° to +31.87°), with 4 observations <−45° shown across a broken y axis and included in all calculations; (B) BlazePose versus Kinovea 2D reference among outputs with target-frame confidence ≥0.3 (n=177, 15 participants overall; bias +5.12°; 95% LoA –11.30° to +21.55°); (C) Kinovea Rater A versus Rater B (n=210, 15 participants; bias +1.15°; 95% LoA –3.51° to +5.81°). The solid dark line is the mean bias, the dashed lines are repeated-measures 95% LoA from within- and between-participant variance components, and the dotted line is zero. These limits are not CIs; CIs for their endpoints are in Table S17 in Multimedia Appendix 1. Points are repeated observations. Pooled lines summarize mixtures of angles or tasks; they do not imply constant bias or uniform coverage across those conditions. Angle- and task-specific limits guide interpretation (Tables 2–4).

Task-specific ICCs were 0.999 for squat, 0.885 for sit-to-stand, and 0.973 for single-leg stance. The sit-to-stand point estimate of 0.885 was less than the a priori descriptive benchmark of 0.90, and its cluster-bootstrap 95% CI was 0.804 to 0.918. Sit-to-stand had the largest interrater MAE (3.13°) and a clinical-flexion bias of +2.65° for Rater A minus Rater B. The lower sit-to-stand ICC may reflect the more gradual transition around peak flexion, which makes the selection of a single target frame less distinct than in squat; this is a plausible explanation rather than a demonstrated mechanism.

### Static Agreement With Goniometer-Set Targets

Across 180 static observations from 15 participants, ICC_2,1_ was 0.874 (cluster-bootstrap 95% CI 0.770 to 0.953), MAE was 10.01°, and RMSE was 15.49° (Table 3; Figure 2A). These differences include uncertainty in target positioning and holding as well as BlazePose estimation and are reported without a universal clinical acceptability cutoff.

Signed error varied by target angle. BlazePose overestimated clinical flexion at 30° (+11.11°) and 60° (+10.18°) and underestimated it at 90° (−4.64°) and 120° (−10.60°). Dispersion was substantially wider at 90° and 120° than at 30° and 60°. This range compression did not support a single fixed correction across the tested angles and did not identify one causal mechanism.

Excluding the 3 static Session 1 videos reprocessed at the lower pose detection threshold changed static ICC from 0.874 to 0.870 and RMSE from 15.49° to 15.58°. This sensitivity analysis quantifies the effect of the protocol deviation; full results are reported in Multimedia Appendix 1 (Table S14).

### Dynamic Conditional Agreement With the Kinovea 2D Reference

Dynamic measurement was available for 177 of 210 (84.3%) observations. The remaining 33 of 210 (15.7%) observations had target-frame confidence <0.3. Availability reached 71 of 75 (95%) observations for squat, 58 of 75 (77%) observations for sit-to-stand, and 48 of 60 (80%) observations for combined single-leg stance. Failures occurred for 5 of 30 (17%) observations on the left and 7 of 30 (23%) observations on the right. Participant counts and distributions are reported in Tables S1 and S5 in Multimedia Appendix 1.

Failures were concentrated in S10 (11/14, 79%), S13 (6/14, 43%), and S12 (5/14, 36%). Kinovea flexion medians for failed versus retained observations were 113.2° versus 131.0° for squat, 89.0° versus 81.0° for sit-to-stand, and 10.2° versus 8.8° for combined single-leg stance. These are descriptive contrasts not evidence of why detection failed; only 4 squat observations failed. Quartiles, ranges, and participant counts are in Table S5 in Multimedia Appendix 1.

Detection performance also varied by acquisition group. Target-frame median confidence and failures were 0.939 and 2 of 84 (2%) observations, respectively, for S01-S06; 0.643 and 14 of 56 (25%) observations, respectively, for S07-S10; and 0.694 and 17 of 70 (24%) observations, respectively, for S11-S15. No recorded device nor encoding variable changed uniquely at S07 or aligned with the persistent later-group decrease; the known 120-to-60 frames-per-second change occurred earlier, between S02 and S03. Because lighting, exact camera geometry, exposure and focus, attire, and background were incompletely recorded, the cause remains unresolved.

Task-specific ICCs were 0.773 (95% CI 0.564 to 0.875; n=71, 15 participants) for squat, 0.632 (95% CI 0.455 to 0.726; n=58, 12 participants) for sit-to-stand, and 0.136 (95% CI −0.078 to 0.482; n=48, 15 participants) for combined single-leg stance (Table 4). Squat and sit-to-stand had high Pearson correlations (*r*=0.885 and *r*=0.896), but their absolute-agreement ICC point estimates were good and moderate, respectively. BlazePose overestimated clinical flexion by +6.17° and +6.43°, respectively. The restricted near-extension range in single-leg stance reduced between-participant variance and made the ICC sensitive to modest error; occlusion, out-of-plane rotation, perspective shortening, side selection, and landmark localization are additional plausible contributors.

Across 177 retained observations, the pooled cross-task ICC was 0.982 (cluster-bootstrap 95% CI 0.967 to 0.992), with bias +5.12° and repeated-measures LoA −11.30° to +21.55° (Figure 2B). This is a secondary descriptive mixture because between-task angle range inflates between-observation variance; it does not imply uniform agreement across tasks.

Participant mean results are provided in Table S18 in Multimedia Appendix 1. These compare the average retained measurements per person and task, not individual observations. The numbers averaged varied because of detection failures. Earlier exploratory participant-by-task summaries in Tables S10-S11 in Multimedia Appendix 1 are retained with their distinct units and unadjusted intervals explicitly identified.

In the all-output stress test, retaining all 210 numeric outputs produced the largest deterioration for squat, for which the ICC decreased from 0.773 to 0.428 and RMSE increased from 8.78° to 25.43°. The sit-to-stand ICC decreased from 0.632 to 0.596, and the combined single-leg stance ICC decreased from 0.136 to 0.119. The pooled ICC decreased from 0.982 to 0.942. These estimates characterize performance without the quality gate and do not replace the conditional primary analysis.

At the stricter target-frame threshold of ≥0.5, availability was 158 of 210 (75.2%) observations; task-specific ICCs were 0.770 (n=70) for squat, 0.670 (n=50) for sit-to-stand, and 0.204 (n=38) for combined single-leg stance (Table S15 in Multimedia Appendix 1). This sensitivity analysis describes the availability-agreement tradeoff and does not establish an optimal threshold.

### Exploratory Repeated-Session Findings

#### Kinovea 2D Reference Across Sessions

Across the 56 repeated task/trial observations collected 21 days to 29 days apart, the pooled Kinovea repeated-session ICC was 0.991 (95% CI 0.985 to 0.995), with an MAE of 5.63° (Table 5). This pooled value reflects the wide range across tasks and repeated observations of only 4 participants. These 4-participant repeated-session data were insufficient to determine whether Kinovea agreement differed by task.

#### BlazePose Across Sessions

Static repeated-session agreement was an ICC of 0.782 (95% CI 0.617 to 0.877; n=48), with Session 2-minus-Session 1 clinical-flexion bias of −7.30° and a wide LoA (−42.65° to +28.04°). The dynamic all-output automated video-minimum stress test yielded an ICC of 0.522 (95% CI 0.174 to 0.757; n=24), an MAE of 34.71°, and an LoA of −82.04° to +119.89°. At least one raw-peak confidence value <0.3 was present for 10 of the 24 dynamic pairs. These values describe an ungated stress test of repeated automated video minima for 4 participants, not time-matched repeated-session validity.

#### Session 2 Conditional Agreement

In Session 2, 21 of the 56 target-frame observations failed the confidence criterion, leaving 35 for conditional agreement analysis. The pooled cross-task ICC among retained observations was 0.969 (95% CI 0.889 to 0.988), with a bias of +8.10° and an MAE of 8.95°. Because this estimate pools tasks and derives from 4 participants after substantial selection, it is presented as an exploratory descriptive result rather than evidence that Session 1 performance was replicated.

#### Between-Method Difference Across Sessions

Among 34 observations valid in both sessions, the ICC for the paired BlazePose-minus-Kinovea clinical-flexion differences was 0.094 (95% CI −0.225 to 0.405). This wide interval from 4 participants is insufficient to support a stable fixed-offset assumption; it is also insufficient to establish that bias instability is a general property of the method. Larger repeated-session samples are required before calibration or longitudinal change interpretations can be evaluated.

### Data Quality and Detection Reproducibility

Across all 342 videos, the descriptive quality-grade distribution was Grade A, 164/342 (48%); Grade B, 21/342 (6.1%); Grade C, 31/342 (9.1%); and Grade D, 126/342 (36.8%; Table S4 in Multimedia Appendix 1). These video-level quality assurance labels did not determine target-frame inclusion, weighting, or statistical inference and are not measures of target-frame detection success.

Detailed detection quality, retest, subject mean, and sensitivity results are provided in Multimedia Appendix 1.

## Discussion

### Principal Findings

Agreement of single-camera BlazePose knee-flexion estimates with the study references depended on task, flexion angle, and detection quality. With measurement availability of 177 of 210 observations (84.3%), task-specific conditional ICC point estimates ranged from good for squat to poor for single-leg stance. The primary cross-sectional CIs now account for repeated observations through participant-cluster resampling, and LoA incorporated within- and between-participant variance. These changes did not remove the uncertainty from only 12 to 15 contributing participants per dynamic task. Static differences changed direction across target angles and included target-setting and holding uncertainty. Kinovea interrater agreement did not establish 3D accuracy, and 4-participant repeated-session findings remained exploratory.

### Kinovea as a 2D Reference

High Kinovea interrater agreement indicates that 2 raters produced similar 2D annotations and reduced 1 source of uncertainty in the BlazePose comparison. It did not establish Kinovea’s accuracy against 3D kinematics or show that every between-method difference originates from BlazePose. Both methods used a single sagittal view and may have shared projection, landmark, and out-of-plane error.

### Task- and Quality-Dependent Dynamic Agreement

Task-specific estimates are more informative than the pooled cross-task ICC because pooling squat, sit-to-stand, and near-extension single-leg stance greatly expanded the angle range. Squat and sit-to-stand showed strong relative association but only good-to-moderate absolute agreement, illustrating why correlation cannot substitute for agreement metrics. In the clinical-flexion space, BlazePose overestimated flexion by approximately 6° for these tasks, and every task-specific LoA interval extended beyond at least one of the descriptive ±10° anchors. If reproduced clinically, this error direction could make recovery appear greater than it is; the healthy sample cannot quantify that clinical consequence.

The conditional analysis also described performance only when target-frame confidence was adequate. Retaining all numeric outputs produced marked deterioration for squat, and failure rates varied by task, participant, and acquisition group. The unresolved confidence decrease after S01-S06 shows that a constant camera model and file format did not guarantee consistent detection. Any future deployment would therefore require explicit measurement availability reporting and a per-recording quality gate rather than relying only on accuracy among successful detections.

### Angle-Dependent Static Error

Static signed error changed from flexion overestimation at 30° and 60° to underestimation at 90° and 120°. The angle-specific signed bias exceeded the descriptive ±5° anchor at 30°, 60°, and 120°, and every angle-specific LoA interval extended beyond at least one of the descriptive ±10° anchors. Together with the overall RMSE, this direction reversal and dispersion did not support a single calibration offset across the tested range. The result was agreement with goniometer-set targets in healthy participants not evidence that BlazePose can replace clinical goniometry. The goniometer was reapplied and reread before every trial but removed for recording and not reapplied afterward. Consequently, target-setting error, rounding, drift during the hold, and goniometer error cannot be separated from BlazePose error; the manufacturer-stated accuracy is not an in-study calibration estimate.

Plausible contributors included mismatch between image-based and palpated anatomical landmarks, perspective shortening, out-of-plane limb rotation, occlusion or crossing limbs, side-selection errors, and amplification of small landmark displacements when segments approach collinearity. These mechanisms are consistent with the observed angle and task dependence but were not isolated experimentally and should not be stated as proven causes.

### Single-Leg Stance: Restricted Range and Plausible Failure Mechanisms

Single-leg stance had poor concurrent agreement. Because the supporting knee occupied a narrow range near full extension, between-participant variance was small, and ICC was especially sensitive to modest measurement error. Occlusion by the nonstance limb, out-of-plane rotation, perspective, and landmark localization may add error in a single lateral view. Exploratory repeated-session findings from 4 participants did not resolve whether this uncertainty was task-related, method-related, or sample-related and did not establish that instability is intrinsic to the task.

### Exploratory Repeated-Session Findings

The repeated-session analyses were based on 4 participants assessed 21 days to 29 days apart and cannot support definitive reliability or bias stability claims. The n=24 dynamic analysis was an ungated, automated, video-minimum stress test not a time-matched measure, and 10 of the 24 pairs included at least one low-confidence raw peak. The ICC for the between-method difference was imprecise and is best interpreted as insufficient evidence for a stable fixed offset not proof of universal bias drift. The 21‐to-29-day interval also permits real within-person change to contribute to disagreement, so measurement error and biological variation cannot be separated here.

### Limitations

The cross-sectional sample was small (n=15) and recruited by convenience from healthy young adults at one university, and no a priori power calculation was performed. The repeated-session subsample contained 4 participants, producing imprecise estimates from repeated observations nested within individuals. Findings cannot be generalized to older adults, knee osteoarthritis, anterior cruciate ligament reconstruction, total knee arthroplasty, or other clinical populations. One readily available iPhone 17 Pro was used throughout to hold hardware constant; equivalence across phones, lenses, frame rates, and device-specific image processing was not tested.

The static comparator was a rounded goniometer-set target rather than an independent measurement of the recorded posture, and the dynamic comparator was Kinovea 2D annotation rather than marker-based 3D kinematics, so absolute biomechanical accuracy remains unknown. We reprocessed 5 static videos at a lower pose-detection threshold, which was a protocol deviation; sensitivity analyses excluding them changed the estimates only slightly. The 33 failures from the 210 dynamic detection observations were unevenly distributed, making the retained-observation agreement estimates conditional on adequate detection. Target-frame confidence decreased after S01-S06 and did not recover in S11-S15; because acquisition conditions were incompletely recorded, the cause could not be identified. Participant-cluster CIs and repeated-measures LoA addressed clustering in the primary cross-sectional analyses, but with only 12 to 15 contributing participants per dynamic task, all interval procedures remained approximate; bootstrap intervals may have had inadequate coverage. The LoA variance model assumes approximately normal differences, common within-participant variance within a stratum, and conditionally independent residual differences. Consecutive repetitions may violate the last assumption. Resampling whole participants preserves the observed dependence but does not validate those distributional assumptions or guarantee future coverage. Across-angle and cross-task pooled LoA are descriptive only. The target-frame cutoff was established during analysis without a documented calibration basis, and sensitivity analyses did not make it a validated deployment threshold. Four-participant repeated-session intervals remained unadjusted and descriptive. Detailed angle-, side-, participant-, and sensitivity-level estimates are exploratory and were not adjusted as multiple confirmatory tests. The study assessed 1 BlazePose model, sagittal-plane angles, and selected task frames only; frontal/transverse motion and temporal variables were not evaluated.

### Clinical Implications and Future Directions

This evidence supports only feasibility or screening-level research use under controlled acquisition with explicit quality checks. It does not support replacing a clinical goniometer, making precise longitudinal treatment decisions, assuming that a fixed calibration is stable, or using the method as an unconditional remote clinical measurement. Because BlazePose overestimated clinical flexion in squat and sit-to-stand, an unverified reading could overstate apparent recovery or mask a residual deficit; measurements that would affect care should be confirmed with an established clinical assessment.

Priorities for future work are larger, prospectively sized, and multisite cohorts; validation against 3D motion capture; testing in relevant clinical populations; direct cross-device evaluation; and controlled experiments that vary camera geometry, lighting, attire, occlusion, and out-of-plane motion. Repeated-session studies should prespecify the estimand, retain participant-level sampling in the analysis, and report both measurement availability and agreement conditional on successful detection.

### Conclusions

Single-camera MediaPipe BlazePose had dynamic measurement availability of 177 of 210 (84.3%) observations: This included 71 of 75 (95%) observations for squat, 58 of 75 (77%) observations for sit-to-stand, and 48 of 60 (80%) observations for single-leg stance. Among the retained observations, the corresponding ICCs for squat, sit-to-stand, and single-leg stance were 0.773, 0.632, and 0.136, respectively with participant-cluster intervals showing substantial uncertainty. Static agreement with goniometer-set targets varied by angle and included uncertainty in the reference positioning. Four-participant repeated-session findings did not establish stable calibration or reliable longitudinal change measurement. These data support further controlled feasibility and screening-level evaluation with per-recording quality checks not replacement of clinical measurement or routine remote clinical decision-making.

## Supplementary material

10.2196/102399 Multimedia Appendix 1 Supplementary detection quality, exploratory repeated-session, participant-mean, sensitivity, and participant-cluster inference analyses.

## Abbreviations

COSMIN: Consensus-Based Standards for the Selection of Health Measurement Instruments
ICC: intraclass correlation coefficient
LoA: limits of agreement
MAE: mean absolute error
RMSE: root mean square error
ROM: range of motion
SEM: standard error of measurement
