# Supplementary material for: Validation of Single-Camera MediaPipe BlazePose for Knee Joint Angle Measurement Across Multiple Functional Tasks: Concurrent Validity, Interrater Reliability, and Exploratory Test–Retest Reliability Study
Source: JMIR Rehabil Assist Technol. 2026 Nov 3;13:e102399. doi: 10.2196/102399 (PMC13730411; doi:10.2196/102399)
Supplement: Supplementary file 1 [file rehab-v13-e102399-s001.docx]

Multimedia Appendix 1

**Supplementary tables for JMIR Rehabilitation and Assistive Technologies manuscript #102399**

All reader-facing angles use clinical knee flexion, with 0° at full extension. All repeated-session analyses are exploratory.

# Dynamic Target-Frame Detection Quality and Descriptive Quality Grades for All Processed Videos

## Table S1. Participant-Level Target-Frame Results

| **Subject** | **Acquisition group** | **Failed/available, n (%)** | **Median target-frame confidence** |
| --- | --- | --- | --- |
| S01 | S01–S06 | 1/14 (7%) | 0.987 |
| S02 | S01–S06 | 0/14 (0%) | 0.934 |
| S03 | S01–S06 | 0/14 (0%) | 0.807 |
| S04 | S01–S06 | 0/14 (0%) | 0.980 |
| S05 | S01–S06 | 1/14 (7%) | 0.976 |
| S06 | S01–S06 | 0/14 (0%) | 0.903 |
| S07 | S07–S10 | 0/14 (0%) | 0.745 |
| S08 | S07–S10 | 0/14 (0%) | 0.884 |
| S09 | S07–S10 | 3/14 (21%) | 0.679 |
| S10 | S07–S10 | 11/14 (79%) | 0.065 |
| S11 | S11–S15 | 3/14 (21%) | 0.654 |
| S12 | S11–S15 | 5/14 (36%) | 0.590 |
| S13 | S11–S15 | 6/14 (43%) | 0.483 |
| S14 | S11–S15 | 0/14 (0%) | 0.938 |
| S15 | S11–S15 | 3/14 (21%) | 0.669 |

## Table S2. Task-Level Target-Frame Failures

| **Task** | **Failed/available, n (%)** | **Retained, n** | **Median target-frame confidence** |
| --- | --- | --- | --- |
| Overall | 33/210 (15.7%) | 177 | 0.868 |
| Squat | 4/75 (5%) | 71 | 0.933 |
| Sit-to-Stand | 17/75 (23%) | 58 | 0.893 |
| SLS left | 5/30 (17%) | 25 | 0.601 |
| SLS right | 7/30 (23%) | 23 | 0.618 |
| SLS combined | 12/60 (20%) | 48 | 0.611 |

## Table S3. Acquisition-Group Target-Frame Results

| **Acquisition group** | **Failed/available, n (%)** | **Median target-frame confidence** |
| --- | --- | --- |
| S01–S06 | 2/84 (2%) | 0.939 |
| S07–S10 | 14/56 (25%) | 0.643 |
| S11–S15 | 17/70 (24%) | 0.694 |

## Table S4. Descriptive Quality Grades for All 342 Processed Videos

| **Grade** | **Videos, n/N (%)** |
| --- | --- |
| A | 164/342 (48%) |
| B | 21/342 (6.1%) |
| C | 31/342 (9.1%) |
| D | 126/342 (36.8%) |

Note. Target-frame confidence is the minimum BlazePose visibility score across the hip, knee, and ankle landmarks at the Kinovea-selected time-matched frame for each dynamic observation. A failed observation had target-frame confidence <0.3. Acquisition-group medians declined after S01–S06 and did not recover in S11–S15. The separate grade panel summarizes all 342 processed videos (static and dynamic, both sessions). Grades A–D are a descriptive quality-assurance summary based on the proportion of frames with confidence ≥0.8: A, ≥80%; B, 50%–79%; C, 20%–49%; and D, <20%. Grades did not determine inclusion, weighting, or inference.

## Table S5. Kinovea Clinical Flexion in Failed and Retained Dynamic Observations

| **Task** | **Status** | **n** | **Participants** | **Median [Q1 to Q3], °** | **Range, °** |
| --- | --- | --- | --- | --- | --- |
| Squat | failed | 4 | 2 | 113.2 [111.0 to 115.1] | 109.5 to 115.5 |
| Squat | retained | 71 | 15 | 131.0 [121.5 to 143.2] | 102.0 to 150.5 |
| STS | failed | 17 | 4 | 89.0 [80.0 to 91.5] | 77.5 to 92.5 |
| STS | retained | 58 | 12 | 81.0 [75.6 to 88.9] | 64.5 to 97.0 |
| SLS | failed | 12 | 7 | 10.2 [4.8 to 12.6] | 2.0 to 16.5 |
| SLS | retained | 48 | 15 | 8.8 [5.5 to 13.6] | 0.0 to 22.5 |

Confidence <0.3 defines failure. Kinovea, not the failed BlazePose output, supplies the flexion values. SLS combines both sides. Differences are descriptive, with repeated observations per participant; no causal attribution or significance test is made.

# Exploratory Participant-Level Repeated-Session Breakdowns

Shared note. All repeated-session analyses are exploratory and include four participants. The observed interval was 21–29 days (median 25 days). Repeated task/trial observations are nested within four participants; confidence intervals therefore do not remove participant-level imprecision. All displayed angles and signed differences use clinical knee flexion, with 0° at full extension. These four-participant F intervals and trial-level LoA are unadjusted, descriptive, and not used for primary cross-sectional inference.

## Table S6. BlazePose Static Repeated-Session Agreement: Session 2 − Session 1 Clinical Flexion

| **Subject** | **n** | **ICC(2,1) [95% CI]** | **MAE (°)** | **Bias (°)** | **RMSE (°)** |
| --- | --- | --- | --- | --- | --- |
| S01 | 12 | 0.472 [−0.059, 0.808] | 27.14 | −24.14 | 36.98 |
| S02 | 12 | 0.980 [0.931, 0.994] | 4.58 | +2.37 | 5.60 |
| S03 | 12 | 0.997 [0.990, 0.999] | 1.49 | +0.62 | 1.82 |
| S04 | 12 | 0.947 [0.131, 0.990] | 8.07 | −8.07 | 9.24 |

## Table S7. Dynamic Repeated-Session All-Output Automated-Video-Minimum Stress Test: Quality Accounting

| **Subject** | **Available pairs, n** | **Both endpoints raw-peak confidence ≥0.3, n** | **At least one endpoint raw-peak confidence <0.3, n** |
| --- | --- | --- | --- |
| S01 | 6 | 1 | 5 |
| S02 | 6 | 4 | 2 |
| S03 | 6 | 5 | 1 |
| S04 | 6 | 4 | 2 |
| Total | 24 | 14 | 10 |

Note. The n=24 dynamic repeated-session analysis used one automated global minimum included angle per video, converted here to clinical flexion, without a raw-peak confidence gate. It was not a Kinovea time-matched peak-flexion estimate. Because 10/24 paired observations had raw-peak confidence <0.3 at one or both sessions, the estimate is presented as an exploratory all-output stress test. This table documents detection quality for the exploratory n=24 stress test reported in Table 5 of the main text.

## Table S8. Kinovea 2D-Reference Repeated-Session Agreement: Session 2 − Session 1 Clinical Flexion

| **Subject** | **n** | **ICC(2,1) [95% CI]** | **MAE (°)** | **Bias (°)** | **RMSE (°)** |
| --- | --- | --- | --- | --- | --- |
| S01 | 14 | 0.990 [0.970, 0.997] | 6.54 | −2.18 | 7.04 |
| S02 | 14 | 0.989 [0.777, 0.998] | 6.11 | +6.04 | 7.53 |
| S03 | 14 | 0.994 [0.979, 0.998] | 4.96 | +3.18 | 6.37 |
| S04 | 14 | 0.991 [0.973, 0.997] | 4.93 | −2.29 | 6.15 |

## Table S9. Session-2 Conditional Agreement: BlazePose − Kinovea 2D-Reference Clinical Flexion

| **Subject** | **Retained/available, n** | **ICC(2,1) [95% CI]** | **MAE (°)** | **Bias (°)** | **RMSE (°)** |
| --- | --- | --- | --- | --- | --- |
| S01 | 6/14 | 0.911 [0.541, 0.987] | 13.81 | +13.81 | 24.71 |
| S02 | 10/14 | 0.991 [0.954, 0.998] | 7.28 | +4.31 | 7.63 |
| S03 | 11/14 | 0.998 [0.953, 1.000] | 3.50 | +3.50 | 4.41 |
| S04 | 8/14 | 0.930 [0.116, 0.989] | 14.87 | +14.87 | 17.43 |

Note. These estimates are conditional on Session-2 target-frame confidence ≥0.3. They are exploratory Session-2 estimates, not independent replication evidence.

# Exploratory Subject-Mean Sensitivity Analyses

## Table S10. Kinovea Inter-Rater Agreement

| **Group** | **n subject means** | **ICC(2,1) [95% CI]** | **MAE (°)** |
| --- | --- | --- | --- |
| Overall subject-by-task | 45 | 0.999 [0.997, 1.000] | 1.26 |
| Squat | 15 | 1.000 [0.999, 1.000] | 0.25 |
| Sit-to-Stand | 15 | 0.908 [0.443, 0.976] | 2.87 |
| SLS | 15 | 0.983 [0.909, 0.995] | 0.65 |

## Table S11. Dynamic BlazePose–Kinovea Agreement

| **Group** | **n subject means** | **ICC(2,1) [95% CI]** | **MAE (°)** |
| --- | --- | --- | --- |
| Overall subject-by-task | 54 | 0.984 [0.966, 0.991] | 6.59 |
| Squat | 15 | 0.816 [0.231, 0.947] | 6.62 |
| Sit-to-Stand | 12 | 0.626 [−0.096, 0.902] | 6.84 |
| SLS left | 14 | 0.321 [−0.258, 0.721] | 5.96 |
| SLS right | 13 | −0.004 [−0.445, 0.498] | 7.02 |

These earlier exploratory summaries average observations within participant or participant-by-task/side cells. They estimate agreement after averaging, not agreement of individual observations. The pooled rows contain multiple cells from the same participant and their conventional F intervals do not account for that dependence; they are descriptive only. Task-specific participant-mean estimates with their exact contributing participant counts are provided in Table S5C. Primary observation-level results instead use participant-cluster inference (Tables 2–4 and S5). Three decimal places are retained.

# Detection-Failure and Protocol-Deviation Sensitivity Analyses

## Table S12. Dynamic All-Output Stress Test Versus the Primary Confidence-Qualified Analysis

| **Group** | **Inclusion rule** | **n/available** | **ICC(2,1) [95% CI]** | **Bias (°)** | **MAE (°)** | **RMSE (°)** |
| --- | --- | --- | --- | --- | --- | --- |
| Squat | Confidence ≥0.3 | 71/75 | 0.773 [0.225, 0.910] | +6.17 | 7.10 | 8.78 |
| Squat | All numeric outputs | 75/75 | 0.428 [0.222, 0.596] | +0.33 | 12.23 | 25.43 |
| Sit-to-Stand | Confidence ≥0.3 | 58/75 | 0.632 [−0.081, 0.872] | +6.43 | 6.68 | 7.59 |
| Sit-to-Stand | All numeric outputs | 75/75 | 0.596 [−0.087, 0.856] | +6.90 | 7.10 | 8.01 |
| SLS combined | Confidence ≥0.3 | 48/60 | 0.136 [−0.150, 0.401] | +2.01 | 7.51 | 12.88 |
| SLS combined | All numeric outputs | 60/60 | 0.119 [−0.136, 0.359] | +1.67 | 7.54 | 12.14 |
| Overall, descriptive | Confidence ≥0.3 | 177/210 | 0.982 [0.952, 0.991] | +5.12 | 7.07 | 9.73 |
| Overall, descriptive | All numeric outputs | 210/210 | 0.942 [0.924, 0.956] | +3.06 | 9.06 | 17.21 |

Note. Bias is BlazePose minus Kinovea clinical flexion at the Kinovea-selected target frame; positive values indicate greater flexion estimated by BlazePose. The primary analysis estimates agreement conditional on adequate target-frame confidence. The all-output analysis is a stress test for deployment without a quality gate; low-confidence numerical outputs may be detection failures rather than valid noisy measurements. The pooled overall ICC is descriptive and may be inflated by the range created by combining heterogeneous tasks. The F-distribution CIs in this sensitivity table are unadjusted descriptive comparators, not the primary cluster-bootstrap intervals. Refer to Table S5A for cluster intervals for the ≥0.3 analysis.

## Table S13. Five Threshold-Rescued Static Trials

| **Trial ID** | **Session** | **Default threshold** | **Rescue threshold** |
| --- | --- | --- | --- |
| S09_static_120deg_2 | Session 1 | 0.5 | 0.3 |
| S10_static_120deg_2 | Session 1 | 0.5 | 0.3 |
| S10_static_30deg_3 | Session 1 | 0.5 | 0.3 |
| S04_retest_static_120deg_2 | Session 2 | 0.5 | 0.3 |
| S04_retest_static_120deg_3 | Session 2 | 0.5 | 0.3 |

Note. Threshold refers to the MediaPipe pose-detection confidence parameter applied during processing; it is distinct from the target-frame confidence ≥0.3 inclusion rule in Table S1. Trial identifiers were confirmed from contemporaneous processing records.

## Table S14. Exclusion Sensitivity for Threshold-Rescued Trials

| **Analysis** | **Rule** | **n** | **ICC(2,1) [95% CI]** | **Bias (°)** | **MAE (°)** | **RMSE (°)** |
| --- | --- | --- | --- | --- | --- | --- |
| Static validity | All trials | 180 | 0.874 [0.834, 0.904] | +1.51 | 10.01 | 15.49 |
| Static validity | Exclude 3 rescued Session-1 trials | 177 | 0.870 [0.830, 0.902] | +1.52 | 10.05 | 15.58 |
| Exploratory static retest | All pairs | 48 | 0.782 [0.617, 0.877] | −7.30 | 10.32 | 19.28 |
| Exploratory static retest | Exclude 2 rescued retest pairs | 46 | 0.766 [0.588, 0.869] | −7.56 | 10.71 | 19.69 |

Note. Static validity bias is BlazePose clinical flexion minus target clinical flexion. Static retest bias is Session 2 minus Session 1 clinical flexion. The small changes in the displayed estimates do not alter the interpretation of the static validity analysis; all repeated-session estimates remain exploratory because only four participants contributed Session-2 data. All F intervals in this protocol-deviation sensitivity table are unadjusted descriptive comparisons; primary Session-1 inference uses Table S5.

## Table S15. Stricter Target-Frame Confidence Threshold of ≥0.5

| **Task** | **Available measurement, n/N (%)** | **ICC(2,1)** | **Bias, °** | **MAE, °** | **RMSE, °** |
| --- | --- | --- | --- | --- | --- |
| Squat | 70/75 (93%) | 0.770 | +6.49 | 6.97 | 8.62 |
| STS | 50/75 (67%) | 0.670 | +5.80 | 6.09 | 6.82 |
| SLS | 38/60 (63%) | 0.204 | +2.53 | 6.19 | 11.80 |
| Overall | 158/210 (75.2%) | 0.984 | +5.32 | 6.50 | 9.01 |

The stricter threshold is a sensitivity analysis, not a calibrated or optimized cutoff. ICCs are descriptive point estimates; the overall row pools heterogeneous tasks. The all-output and ≥0.3 comparisons are in S4A.

# Participant-Cluster Inference

Primary cross-sectional intervals use 10,000 participant-cluster resamples (master seed 20260923). All paired observations from each sampled participant are retained; trials are not independently resampled. Only participants with retained pairs contribute to conditional agreement. Stable analysis-specific seeds and exact resampling rules are documented below. No interval method eliminates the uncertainty from the small number of participants.

## Table S16. Observation-Level ICC and Bias With Cluster-Bootstrap 95% CIs

| **Analysis** | **n** | **Participants** | **ICC(2,1) [95% CI]** | **Bias [95% CI], °** |
| --- | --- | --- | --- | --- |
| Static overall | 180 | 15 | 0.874 [0.770 to 0.953] | +1.51 [−1.47 to +4.05] |
| Dynamic Squat | 71 | 15 | 0.773 [0.564 to 0.875] | +6.17 [+3.31 to +9.07] |
| Dynamic STS | 58 | 12 | 0.632 [0.455 to 0.726] | +6.43 [+4.25 to +8.60] |
| Dynamic SLS | 48 | 15 | 0.136 [−0.078 to 0.482] | +2.01 [−1.74 to +7.02] |
| Dynamic pooled | 177 | 15 | 0.982 [0.967 to 0.992] | +5.12 [+2.79 to +7.71] |
| Inter-rater pooled | 210 | 15 | 0.999 [0.998 to 0.999] | +1.15 [+0.63 to +1.68] |
| Inter-rater Squat | 75 | 15 | 0.999 [0.997 to 0.999] | +0.09 [−0.08 to +0.27] |
| Inter-rater STS | 75 | 15 | 0.885 [0.804 to 0.918] | +2.65 [+1.39 to +3.99] |
| Inter-rater SLS | 60 | 15 | 0.973 [0.946 to 0.984] | +0.58 [+0.23 to +0.98] |

ICC, bias, MAE, and RMSE point estimates retain the original observation weighting. Pooled rows are secondary descriptive mixtures. Static angle-specific ICCs are not estimated because the target is constant within each angle. Bias is BlazePose minus reference, or Rater A minus Rater B for inter-rater comparisons. The bootstrap conditions on the studied methods and eligible participant sample.

## Table S17. Repeated-Measures LoA and Cluster-Bootstrap CIs for Each Endpoint

| **Analysis** | **Former unadjusted LoA, °** | **Lower RM LoA [95% CI], °** | **Upper RM LoA [95% CI], °** |
| --- | --- | --- | --- |
| Static overall | −28.79 to +31.81 | −28.85 [−43.54 to −14.65] | +31.87 [+20.16 to +41.16] |
| Static 30deg | −1.45 to +23.67 | −1.73 [−5.67 to +4.57] | +23.94 [+19.23 to +27.24] |
| Static 60deg | −2.37 to +22.73 | −2.66 [−8.56 to +4.03] | +23.03 [+16.10 to +28.25] |
| Static 90deg | −39.15 to +29.86 | −39.36 [−63.07 to −7.17] | +30.07 [+5.48 to +40.22] |
| Static 120deg | −39.69 to +18.48 | −39.71 [−64.94 to −12.41] | +18.50 [−3.55 to +35.22] |
| Dynamic Squat | −6.17 to +18.50 | −6.45 [−10.30 to −1.55] | +18.78 [+13.07 to +22.86] |
| Dynamic STS | −1.56 to +14.42 | −1.82 [−5.05 to +1.66] | +14.67 [+10.54 to +17.34] |
| Dynamic SLS | −23.18 to +27.20 | −23.42 [−29.12 to −13.44] | +27.44 [+11.42 to +40.77] |
| Dynamic pooled | −11.15 to +21.39 | −11.30 [−14.41 to −7.34] | +21.55 [+14.93 to +27.75] |
| Inter-rater pooled | −3.49 to +5.79 | −3.51 [−4.26 to −2.66] | +5.81 [+4.29 to +7.08] |
| Inter-rater Squat | −1.24 to +1.43 | −1.25 [−1.59 to −0.91] | +1.43 [+1.04 to +1.80] |
| Inter-rater STS | −3.67 to +8.98 | −3.78 [−5.46 to −1.68] | +9.08 [+6.63 to +10.84] |
| Inter-rater SLS | −1.87 to +3.04 | −1.88 [−2.16 to −1.49] | +3.05 [+2.37 to +3.69] |

RM=repeated measures. Limits are bias±1.96√(within-participant variance + between-participant variance), using the unequal-replicate variance-components method of Bland and Altman (2007; main-text reference 16). Negative estimated between-participant components are truncated at zero. The CIs describe uncertainty in each endpoint, not the range of individual differences. Pooled static limits mix target angles with different biases; pooled dynamic and inter-rater limits mix tasks. These summaries do not imply constant bias or uniform future coverage. The method assumes approximately normal differences, common within-participant variance within strata, and conditionally independent residual differences. Few participants limit interval precision, and consecutive repetitions may violate the conditional-independence assumption.

## Table S18. Supplementary Agreement of Participant Mean Measurements

| **Analysis** | **Participants** | **Observations averaged per person** | **ICC(2,1) [F 95% CI]** | **Mean difference, °** |
| --- | --- | --- | --- | --- |
| dynamic Squat | 15 | 2 to 5 | 0.816 [0.231 to 0.947] | +5.82 |
| dynamic STS | 12 | 3 to 5 | 0.626 [−0.096 to 0.902] | +6.60 |
| dynamic SLS | 15 | 1 to 4 | 0.087 [−0.403 to 0.553] | +3.45 |
| interrater Squat | 15 | 5 to 5 | 1.000 [0.999 to 1.000] | +0.09 |
| interrater STS | 15 | 5 to 5 | 0.908 [0.443 to 0.976] | +2.65 |
| interrater SLS | 15 | 4 to 4 | 0.983 [0.909 to 0.995] | +0.58 |

Each row has one paired mean per participant and task; the conventional F intervals apply to that independent-pair analysis. The estimand is agreement after averaging the available observations, not single-observation agreement or the reliability of a fixed number of repeats. Different participant weights explain why mean differences can differ from observation-weighted primary biases. SLS combines left and right observations before calculating one participant mean.

## Table S19. Static Bias Based on Participant Means Within Each Target Angle

| **Target** | **Participants** | **Bias, °** | **SD of participant mean bias, °** | **t-based 95% CI for bias, °** |
| --- | --- | --- | --- | --- |
| 30° | 15 | +11.11 | 6.43 | +7.55 to +14.67 |
| 60° | 15 | +10.18 | 6.54 | +6.56 to +13.80 |
| 90° | 15 | −4.64 | 12.66 | −11.65 to +2.37 |
| 120° | 15 | −10.60 | 8.87 | −15.51 to −5.69 |

Three trials are averaged within each participant and target. Each angle-specific row contains 15 independent participants; the four angles are not independent samples of 15 different people. No pooled F interval treating the 60 participant-by-angle cells as independent is used.

Reproducibility: for each analysis label, the seed is the unsigned little-endian integer represented by the first eight bytes of SHA-256 of the UTF-8 string "20260923:<analysis_label>". NumPy default_rng draws eligible participant indices with replacement; 2.5th and 97.5th percentiles use linear interpolation. Labels used in S5A are static_Overall; dynamic_Squat, dynamic_STS, dynamic_SLS, dynamic_Overall; and interrater_Overall, interrater_Squat, interrater_STS, interrater_SLS. Static strata use static_30deg, static_60deg, static_90deg, and static_120deg. Software versions are reported in the main Methods. The analysis script and source summaries are available under the stated Data Availability policy.
